# Developmental defects of the enamel and its impact on the oral health quality of life of children resident in Southwest Nigeria

**DOI:** 10.1186/s12903-018-0622-3

**Published:** 2018-09-27

**Authors:** Morenike Oluwatoyin Folayan, Nneka Maureen Chukwumah, Bamidele Olubukola Popoola, Dada Oluwaseyi Temilola, Nneka Kate Onyejaka, Titus Ayo Oyedele, Folake Barakat Lawal

**Affiliations:** 1 Department of Child Dental Health, Obafemi Awolowo University, Ile-Ife, Nigeria; 2 Department of Child Dental Health, Obafemi Awolowo University Teaching Hospitals’ Complex, Ile-Ife, Nigeria; 3 University of Benin Teaching Hospital, Benin City, Edo State Nigeria; 4 University of Ibadan, Ibadan, Nigeria; 5 University of Nigeria, Enugu, Nigeria; 6 Department of Surgery, Benjamin Carson, Snr, School of Medicine, Babcock University, Ilisan-Remo, Ogun State Nigeria; 7 Dental Department, Babcock University Teaching Hospial, Ilisan-Remo, Ogun State Nigeria

**Keywords:** Enamel, Hypoplasia, Hypomineralisation, Children, Quality of life, Nigeria

## Abstract

**Background:**

Developmental defects of the enamel (DDE) increase the risk for diseases that impact negatively on the quality of life. The objective of this study was to compare the oral health quality of life of children with molar-incisor-hypomineralisation (MIH) and enamel hypoplasia; and assess if caries worsened the impact of these lesions on the quality of life.

**Methods:**

This study recruited 853 6 to 16-years-old school children. They filled the Child-OIDP questionnaire. The MIH, enamel hypoplasia, caries and oral hygiene status was assessed. Poisson regression was used to determine the impact of MIH and enamel hypoplasia on the oral health quality of life, after adjusting for the effect of sex, age, socioeconomic class, oral hygiene and caries status.

**Results:**

The prevalence of MIH and enamel hypoplasia was 2.9% and 7.6% respectively. There was no significant difference in the mean child-OIDP scores of children with or without MIH (*p* = 0.57), children with or without enamel hypoplasia (*p* = 0.48), and children with enamel hypoplasia with and without caries (*p* = 0.30). Children with enamel hypoplasia and caries had worse outcomes for speaking (*p* = 0.01). Children with middle (AOR: 2.74; 95% CI: 1.60–4.67; *P* < 0.01) and low (AOR: 1.75; 95% CI: 1.04–2.95; *p* = 0.03) socioeconomic status, and those with caries (AOR: 2.02; 95% CI: 1.26–3.22; *p* = 0.03) had their oral health quality of life negatively impacted.

**Conclusion:**

MIH and enamel hypoplasia had no significant impact on the overall oral health quality of life of children resident in southwestern Nigeria. However, children with caries and those from middle and low socioeconomic classes had poorer oral health quality of life.

## Background

Developmental defects of the teeth are caused by complex interactions between genetic and environmental factors that affect the structure of the enamel during its formation [1]. Developmental defects of the enamel (DDE) can be classified into two distinct categories: those that affect the quality (hypomineralisation) and those that affect the quantity (hypoplasia) of the enamel [2].

One of the most studied forms of hypomineralised enamel is molar hypomineralisation [MIH].

The lesion arises from the disruption of ameloblasts during mineralization and maturation phase of the enamel, thereby giving rise to defective quality of the enamel [3]. The defect affects one to four first permanent molars, and is frequently associated with the affected permanent incisors [4]. A similar lesion has been reported in second primary molars [5, 6]. The defect caused by MIH appears in white, yellow or brown colour, reflecting the hypomineralised nature, while hypoplasia presents as an area of reduced thickness of the enamel in form of pits, grooves, and bands.

The prevalence of MIH ranges between 3.5 and 40.2% [7]. The aetiological factors remain largely unknown, though multiple systemic aetiological factors have been suggested, including the possibility of genetic predisposition [8]. It is an important risk factor for caries in the permanent dentition [9]. The associated post-eruptive breakdown due to soft and porous enamel is associated with tooth sensitivity, disfiguration and rapid plaque retention [3]. Caries [10], dentine sensitivity [11] and poor aesthetics [12] - morbidities associated with MIH – impact negatively on the quality of life of affected individuals. There are however, no studies highlighting the negative impact of MIH on the quality of life, though Arrow [13] reported no impact of enamel defect on the first molars on the quality of life of affected children in Australia.

Unlike MIH whose aetiology remains unclear, enamel hypoplasia is known to be a quantitative defect which results in the formation of clinically visible, localized or generalized pits and grooves on the affected tooth [14]. A high prevalence of the lesion has been observed in children from developing countries [15], children with chronic or acute malnutrition [15], and children with very low birth weight [16]. Enamel hypoplasia has also been associated with increased risk for poor aesthetics [17], caries [18] and poor oral hygiene [19]. We found no study discussing the effect of enamel hypoplasia specifically, on the quality of life of affected individuals. However, Vargas-Ferreira and Ardenghi [17] reported no impact of developmental dental defects on the overall quality of life of affected children, though it caused significant functional limitation.

MIH and enamel hypoplasia both cause discoloration. While discoloration of the posterior teeth may have no significant impact on the psychology of patients, it is expected that discoloration of the anterior teeth – which both MIH and enamel hypoplasia could affect – may negatively impact on the quality of life. Discolorations and aesthetic concerns associated with enamel hypoplasia may however be more severe than that associated with MIH. This increases the possibility of enamel hypoplasia having worse impact on the quality of life when compared with MIH. There is however, no studies to verify this hypothesis.

Past studies conducted in Nigeria showed a high prevalence of MIH [20, 21] and enamel hypoplasia [22, 23]. The prevalence of MIH ranged between 9.7% and 17.7% [20, 21], while that of enamel hypoplasia ranged between 0.13% and 3.6% in the permanent dentition [22–26] and 2.3% to 4.0% in the primary dentition [23, 27]. Co-existence of MIH and enamel hypoplasia had also been described [28]. These lesions were also associated with co-morbidities that affect oral health quality of life [29, 30]. Nigeria therefore provides a good environment to study the impact of MIH and enamel hypoplasia on the oral health quality of life of children. We, therefore, assessed the impact of MIH and enamel hypoplasia on the oral health quality of life of children resident in southwestern Nigeria. We also compared the quality of life of children with DDE with those without DDE and assessed if caries worsened the outcome of DDE on their quality of life.

## Methods

### Study design

This was a cross-sectional study. Study participants were 6 to 16 years old children and adolescents living in Ile-Ife and Ibadan, Nigeria. Children aged six years were included in the study population because of the possibility of having enamel hypoplasia in the primary dentition [23]. Also, children in the study environment erupt their first permanent molars early: the first permanent mandibular molars are out by 5.68 ± 1.21 years in boys and girls and the first permanent maxillary molars are out by 5.95 ± 0.96 years in girls and 6.15 +  0.93 years in boys [31].

Participants were recruited from public and private secondary schools to ensure representation of all cadres of the socioeconomic strata in the study sample. Socioeconomic status has been associated with the presence of enamel hypoplasia [32]. In Nigeria, children who attend public schools are majorly from the lower socioeconomic strata, while most of those who attend private schools are from the upper socioeconomic strata [33]. Children are taught in English. Public communication is also in English.

### Study location

One of the two study locations was Ife Central Local Government Area (LGA), a sub-urban town in south-western Nigeria. The last census showed that the population of the LGA was 138,818, with about 14,000 (10%) being children [34]. The study site also hosts oral health clinics thereby making it possible to refer screened pupils with lesions for oral health care. The second study site was Ibadan, an urban town in southwestern Nigeria. Ibadan the capital city of Oyo State, has a population of about two million, 36% of which are children [35].

### Sample size and sampling procedure

The sample size was powered to determine the prevalence of DDE based on the assumption that prevalence of the lesion was 50%. The sample size was calculated using the Cochran formula [36]. This gave a minimum sample size of 384. The sample size was increased by 10% to account for attrition, giving a required sample size of 422 study participants.

A multi-phase sampling method was employed. The first stage involved selection of schools using a simple random sampling technique from a list of 107 registered public and private primary and secondary schools in the Ife-Central LGA and a list of 102 primary and secondary schools in Ibadan North-East LGA. The list was obtained from the Local School Authority. The schools were stratified into public and private schools. Eight (three public and five private) of the 107 schools in Ile-Ife and 12 of the 102 schools in Ibadan were randomly selected by picking blindly from pot containing the names of the schools. A ratio that ensured proportionate representation of both public and private schools in the LGA was used to guide the number of schools to be selected.

The class registration list for each school was then used to determine the specific classes to participate in the study. In each school children aged 6 to 16 years eligible for study participation were enlisted from the study register. Twenty two students were then randomly selected by balloting from the list of eligible students. The recruited students were then met, introduced to the study and given consent forms for their parents to fill. All children eligible to participate in the study who were in school on the day of examination and who had parental consent to participate in the study were enrolled. Children with special care needs were excluded from the study because of an increased likelihood of their having poorer oral health quality of life [37].

### Child Oral impact on daily performance (child-OIDP) assessment

The English version of the child version of Oral Impact on Daily Performance (Child-OIDP) questionnaire [38] validated by Chukwumah et al. [30] was used to collect data on oral health quality of life. Trained interviewers administered the questionnaire. It generated information on age, sex, father’s occupation, mother’s level of education and the oral health quality of life for each child.

### Clinical examination

#### Study procedure

All eligible participants were examined in the classroom in the presence of a teacher. Each child was seated on a chair facing the window to ensure a natural source of light for intra-oral viewing. The dental mirror was used to further provide illumination of the tooth surfaces through reflection of light and sunrays. To ensure privacy, a corner was created in each classroom to conduct the clinical examination.

#### Diagnosis of caries

Caries was diagnosed using the World Health Organisation Oral Health Survey recommendations [39]. Each tooth was examined for dental caries using the ball tipped WHO dental explorer. Caries status was assessed using the Decayed Missing and Filled Teeth (dmft/DMFT) index. Decayed tooth (d/D) were defined as any tooth whose crown had an unmistakable cavitation on the pits or fissures, or tooth surface, or a filled crown with decay. This implies that caries was diagnosed only when there was dentine involvement. The filled tooth (f/F) was defined as a filled crown with no decay. The missing tooth (m/M) was defined as a tooth extracted due to caries. To arrive at a dmft/DMFT score, the number of teeth with caries, number of extracted teeth due to caries, and the number of teeth with fillings or crowns were summed together [40]. For the purpose of analysis, caries status was further divided into caries present (when dmft/DMFT was 1 or greater) or absent (when dmft/DMFT was 0). Enamel defects were differentiated from carious lesions by their clinical appearance and locations (usually not related to gingival margins or occlusal fissures) [41].

#### Measuring oral hygiene status

The components of the OHI-S, the Debris Index and Calculus Index, were obtained based on six numerical determinations representing the amount of debris or calculus found on the surfaces of index teeth namely: 11, 16, 26, 31, 36, 46 [42]. Debris and calculus scores were totaled and divided by the number of surfaces scored. Scores were graded as 0.0–1.2 = Good oral hygiene, 1.3–3.0 = Fair oral hygiene and > 3.1 = Poor oral hygiene.

#### Diagnosis of developmental defects of the enamel

All the teeth were examined wet after debris had been removed with the use of a piece of gauze after the OHI-S assessment. Each surface of the permanent first molar and incisor was screened for demarcated white, yellow or brown opacities, greater or equal to 2 mm in diameter based on European Association of Peadiatric Dentistry’s diagnostic criteria for MIH [43]. All the teeth were also examined for enamel hypoplasia. A diagnosis of enamel hypoplasia was made when there was evidence of deficiency in enamel formation seen clinically as localized or generalized pits and grooves on the surfaces of teeth. Both primary and permanent dentitions were examined.

#### Calibration

The two examiners were trained Paedodontists who had been practicing for a minimum of 10 years. They both undertook a series of calibration exercises to ensure the validity of their diagnosis of dental anomalies; the WHO criteria for the diagnosis of caries [39] and the OHI-S index described by Green and Vermillion [42]. The examiners had several sessions reviewing clinical photographs and repeated practice on examination of lesions, using clinical photographs. The kappa scores for the inter-examiner reliability score for caries, oral hygiene status, MIH and enamel hypoplasia were 0.96, 0.95, 0.50 and 0.72 respectively.

### Self report of oral health condition

A checklist of 17 oral conditions was used to assess the child’s experience of oral disease in the previous 3 months. Respondents were instructed to answer “yes” if they had experienced any of the disease conditions in the previous 3 months, and “no” if they had not. Oral conditions were described to the children. The 17 checklist conditions were toothache; sensitive tooth; tooth decay or cavity; loss of primary tooth; tooth space (due to non-erupted permanent tooth); fractured permanent tooth; abnormal colour, shape or size of tooth; abnormal position of tooth (e.g. crooked or projecting, gapped); bleeding gums; swollen gums; calculus; oral ulcers; bad breath; deformity of mouth or face (e.g. cleft lip, cleft palate); erupting permanent tooth; and missing permanent tooth.

The impact of oral hygiene, caries and presence of DDE on the child’s daily performance using the child-OIDP were also assessed. The child-OIDP assessed the impact of caries on eating, speaking, tooth cleaning, relaxing, emotional status, smiling, studying and social contact. If an impact was reported, the child was asked to grade the severity of the impact on daily life performance in each of the eight items using a four-point Likert-like scale with scores ranging from 0 to 3. A score of 0 meant no impact, 1 meant mild impact, 2 meant moderate impact and 3 meant severe impact.

Children indicated the frequency of an oral health problem by assigning a score from 0 to 3 to each of the items for which they indicated an oral lesion impacted their daily life function. A score of 0 meant no event occurred, 1 meant the event occurred once or twice per month, 2 meant the event occurred once or twice per week and 3 meant the event occurred three or more times per week. The oral impact score for each of the eight daily life performance items was obtained by multiplying the severity scores by the frequency scores. Scores ranged from 0 to 9 per item. The overall impact score was the sum of all eight items (ranging from 0 to 72).

### Measure of socioeconomic status

Socioeconomic status was assessed with a multiple item index, combining the mother’s level of education with the father’s occupation [44]. Each child was allocated into one of five social classes (I–V), with V being the lowest. For ease of analysis, three socioeconomic status groups were established: high (children from the upper and upper middle status), middle (children from the middle status) and low (children from the lower middle and lower status) class.

### Data analysis

Data was analysed using the statistical package for social sciences (SPSS) version 17.0. Children were categorized as having or not having MIH, and having or not having enamel hypoplasia. Differences in the item and mean Child-OIDP scores of children with and without MIH; and children with and without enamel hypoplasia were determined respectively. Also, differences in the Child-OIDP scores of children with MIH and enamel hypoplasia who had or did not have caries were also determined respectively. Also, children with MIH were matched with children without any lesion; and children with enamel hypoplasia were matched with children without any lesion. The match was by age, sex and socioeconomic status. Differences in the Child-OIDP scores of matched children were also determined. The McNemar chi square test was used to assess the differences in the mean Child-OIDP scores.

Logistic regression was conducted to determine the impact of the presence of MIH and enamel hypoplasia on the oral health quality of life. Study participants where dichotomized to ‘no impact’ (when the Child-OIDP was zero) and ‘impacted’ (when the Child-OIDP ranged from 1 to 72). Adjustment was made for factors that increased the risk for MIH and enamel hypoplasia in the study environment: socio-economic status [45], caries [46] and poor oral hygiene [47]. Also, adjustment was made for oral hygiene status since MIH and enamel hypoplasia both increase the risk for poor oral hygiene, and oral hygiene status can affect the quality of life [9]. Age and gender were also included in the logistic regression model. The goodness of fit for the chi square tests and logistic regression was assessed using the Cox and Snell R square and the Nagelkerke R square. The model was considered to have a good fit if the tests were not statistically significant. A 95% confidence interval was set to confirm if a relationship truly existed within or between variables. The statistical significance level was set at *P* < 0.05.

### Ethical considerations

Ethical approval for this study was obtained from the Obafemi Awolowo University Teaching Hospitals’ Complex Ethics and Research Committee (IRB/IEC/00004553) and the Oyo State Ministry of Health ethical review committee (AD13/479/649). Permission was also obtained from the State Ministry of Education in Ile-Ife and the principals of participating schools. Prior to commencement of the study, informed consent forms were sent to the parents to get their approval prior to recruitment of study participants. Children 12 years and older gave assent for study participation. Students who required treatment were informed and issued a referral letter. No financial compensation was paid for study participation.

## Results

### Socio-demographic profile of study participants

Table 1 highlights the socio-demographic profile of the study participants. Eight hundred and fifty three participants were recruited for the study. This included 438 (51.3%) males, 428 (50.6%) children aged 6–9 years and 469 (55.0%) children with low socioeconomic status.

**Table 1:** Profile of study respondents (*N* = 853)

Variables	MIH present *N* = 25 n (%)	Enamel hypoplasia present *N* = 65 n (%)	Total *N* = 853n (%)
Age (years)			
6–9	20 (80.0)	22 (33.8)	428 (50.2)
10–13	0 (0.0)	32 (49.2)	302 (35.4)
14–16	5 (20.0)	11 (17.0)	123 (14.4)
*P*- value	0.001	0.02	
Sex			
Male	10 (40.0)	31 (47.7)	438 (51.3)
Female	15 (60.0)	34 (52.3)	415 (48.7)
*P*- value	0.25	0.31	
Socio-economic status			
High SES	12 (48.0)	13 (20.0)	241 (28.3)
Middle SES	5 (20.0)	6 (9.2)	143 (16.8)
Low SES	8 (32.0)	46 (70.8)	469 (55.0)
*P*- value	0.05	0.03	
Caries status			
Caries present	1 (4.0)	10 (15.4)	102 (12.0)
Caries absent	24 (96.0)	55 (84.6)	751 (88.0)
*P*- value	0.21	0.38	
Oral hygiene Status			
Good	5 (20.0)	19 (29.2)	183 (21.5)
Fair	19 (76.0)	40 (61.5)	600 (70.3)
Poor	1 (4.0)	6 (9.2)	70 (8.2)
*P*- value	0.71	0.24	

### Developmental defects of the enamel profile of study participants

Table 1 also highlights the profile of the study participants with DDE. Twenty five (2.9%) children had MIH while 65 (7.6%) children had enamel hypoplasia. One (0.1%) child had both MIH and enamel hypoplasia. Significantly more 6–9 years old children (*p* = 0.001) and children with high socioeconomic status (*p* = 0.05) had MIH. Also, significantly more 10–13 years old children (*p* = 0.02) and children with low socio-economic status (*p* = 0.03) had enamel hypoplasia.

### Caries profile of study participants

Table 1 also highlights the caries profile of the study participants. One hundred and two (10.2%) children had caries. Of these, 1 (1.0%) child had MIH while 10 (9.8%) children had enamel hypoplasia. There was no significant difference in the number of children with and those without caries who had MIH (*p* = 0.21) and those who had enamel hypoplasia (*p* = 0.38). The prevalence of caries was higher in children with enamel hypoplasia when compared with children who had MIH (*p* = 0.01).

### Oral hygiene profile of study participants

Table 1 also highlights the oral hygiene profile of the study participants. One hundred and eighty-three (21.5%) children had good oral hygiene while 70 (8.2%) children had poor oral hygiene. There was no significant difference in the oral hygiene status of children with MIH (*p* = 0.71) and children with enamel hypoplasia (*p* = 0.24).

### Effect of developmental dental anomalies on the quality of life of study participants

Table 2 shows the mean child-OIDP scores of children with DDE. There was no significant difference in the mean child-OIDP scores of children with or without MIH (*p* = 0.57) and the mean child-OIDP scores of children with or without enamel hypoplasia (*p* = 0.48). There was also no significant difference in the mean child-OIDP scores of children with and without MIH, and children with and without enamel hypoplasia in the eight items (eating, speaking, contact, schooling, smiling, emotion, relaxing and cleaning) examined.

**Table 2:** Mean child-OIDP scores of study participants with developmental enamel defects (*N* = 853)

Characteristics	Eating mean ± SD	Speaking mean ± SD	Contact mean ± SD	School mean ± SD	Smiling mean ± SD	Emotion mean ± SD	Relaxing mean ± SD	Cleaning mean ± SD	C-OIDP mean ± SD
Molar Incisor hypomineralisation (MIH)									
MIH present	0.40 ± 1.29	0.12 ± 0.60	0.00 ± 0.00	0.00 ± 0.00	0.20 ± 0.71	0.00 ± 0.00	0.12 ± 0.60	0.00 ± 0.00	0.68 ± 3.01
MIH absent	0.44 ± 1.40	0.11 ± 0.75	0.01 ± 0.12	0.03 ± 0.36	0.05 ± 0.50	0.02 ± 0.26	0.04 ± 0.49	0.41 ± 1.39	1.09 ± 3.58
*P*-value	0.88	0.96	0.76	0.72	0.14	0.68	0.44	0.14	0.57
Enamel hypoplasia									
Enamel hypoplasia present	0.45 ± 1.44	0.17 ± 0.84	0.00 ± 0.00	0.00 ± 0.00	0.05 ± 0.37	0.00 ± 0.00	0.05 ± 0.37	0.68 ± 1.85	1.38 ± 3.67
Enamel hypoplasia absent	0.44 ± 1.40	0.11 ± 0.73	0.01 ± 0.12	0.03 ± 0.37	0.05 ± 0.52	0.02 ± 0.27	0.04 ± 0.51	0.37 ± 1.32	1.06 ± 3.55
*P*-value	0.98	0.52	0.62	0.55	0.91	0.49	0.98	0.09	0.48

### Difference in the quality of life of study participants with and without MIH

Table 3 compares the mean item and composite child-OIDP scores of children with and without MIH matched for sex, age and socioeconomic status. There was also no significant difference in any of the eight items (eating, contact, schooling, smiling, emotion, relaxing and cleaning) examined between children with and without MIH. There was also no difference in the mean child-OIDP scores of children with and without MIH (*p* = 0.86). The Goodness of fit statistics showed that chi square test = 32.00 (*P* < 0.001), indicating poor model fit.

**Table 3:** Mean child-OIDP scores of study participants with and without molar incisor hypomineralization (MIH) matched for age, sex and socioeconomic status (*n* = 50)

Item	MIH present *n* = 25 Mean ± SEM	MIH absent *n* = 25 Mean ± SEM	*P*-value
Eating	0.12 ± 0.09	0.12 ± 0.09	1.00
Speaking	0.00 ± 0.00	0.00 ± 0.00	–
Cleaning	0.12 ± 0.12	0.08 ± 0.08	0.78
Relaxing	0.00 ± 0.00	0.00 ± 0.00	–
Emotion	0.00 ± 0.00	0.00 ± 0.00	–
Smiling	0.00 ± 0.00	0.00 ± 0.00	–
School	0.00 ± 0.00	0.00 ± 0.00	–
Contact	0.00 ± 0.00	0.00 ± 0.00	–
Child OIDP	0.24 ± 0.14	0.2 ± 0.16	0.86

### Difference in the quality of life of study participants with and without enamel hypoplasia

Table 4 compares the mean item and composite child-OIDP scores of children with and without enamel hypoplasia matched for sex, age and socioeconomic status. There was also no significant difference in any of the eight items (eating, contact, schooling, smiling, emotion, relaxing and cleaning) examined between children with and without enamel hypoplasia. There was also no difference in the mean child-OIDP scores of children with and without enamel hypoplasia (*p* = 0.96). The Goodness of fit statistics showed that chi square test = 22.43 (*P* < 0.001), indicating poor model fit.

**Table 4:** Mean child-OIDP scores of study participants with and without enamel hypoplasia matched for age, sex and socioeconomic status (*n* = 130)

Item	Hypoplasia present *n* = 65 Mean ± SEM	Hypoplasia absent *n* = 65 Mean ± SEM	*P*-value
Eating	0.58 ± 1.19	0.43 ± 1.12	0.49
Speaking	0.14 ± 0.10	0.29 ± 0.13	0.34
Cleaning	0.74 ± 0.22	0.68 ± 0.23	0.85
Relaxing	0.03 ± 0.02	0.11 ± 0.08	0.33
Emotion	0.03 ± 0.02	0.00 ± 0.00	0.16
Smiling	0.03 ± 0.02	0.05 ± 0.05	0.76
School	0.00 ± 0.00	0.03 ± 0.03	0.32
Contact	0.00 ± 0.00	0.00 ± 0.00	–
Child OIDP	1.55 ± 0.40	1.58 ± 0.42	0.96

### Factors that impacted on the quality of life of study participants

Table 5 shows the outcome of the logistic regression analysis to determine factors that impacted on the quality of life of study participants. Children from the middle (AOR: 2.74; 95% CI: 1.60–4.67; *P* < 0.01) and low (AOR: 1.75; 95% CI: 1.04–2.95; *p* = 0.03) socioeconomic classes had increased odds of having their quality of life negatively affected when compared with children with high socio-economic status. MIH (*p* = 1.00) and enamel hypoplasia (*p* = 1.00) had no significant impact on the quality of life of the children. Having one or more DDE had no significant impact on the quality of life of the children (*p* = 1.00). The Goodness of Fit for the logistic regression was 0.051 for the Cox and Snell R square test, and 0.08 for the Nagelkerke R square test indicating a poor fit.

**Table 5:** Logistic regression analysis of factors that had impact on the quality of life of children with developmental dental anomalies (N = 853)

Variables	Mean C-OIDP scores	Had impact *N* = 176	Had no impact *N* = 677	Adjusted OR	95% C.I	*P*- value
Age						
6–9	0.68 ± 2.22	68 (38.6)	360 (53.2)	1.00	–	–
10–13	1.57 ± 4.18	81 (46.0)	221 (32.6)	0.91	0.52–1.60	0.75
14–16	1.34 ± 5.23	27 (15.4)	96 (14.2)	0.76	0.46–1.27	0.29
Sex						
Male	1.14 ± 4.23	88 (50.0)	350 (51.7)	1.00	–	–
Female	1.04 ± 2.71	88 (50.0)	327 (48.3)	0.91	0.65–1.27	0.60
Presence of enamel hypoplasia						
Enamel hypoplasia present	1.55 ± 3.21	18 (10.2)	47 (6.9)	1.00	–	–
Enamel hypoplasia absent	1.05 ± 3.60	158 (89.8)	630 (93.1)	0.00	0.00–0.00	1.00
Presence of developmental defect of the enamel						
No DDE	1.08 ± 3.65	155 (20.3)	609 (79.7)	1.00	–	–
1 or 2 DDE	1.20 ± 2.83	21 (23.6)	68 (76.4)	0.00	0.00–0.00	1.00
Presence of MIH						
MIH present	0.24 + 0.72	3 (1.7)	22 (3.2)	1.00	–	–
MIH absent	1.12 + 3.62	173 (98.3)	655 (96.8)	0.00	0.00–0.00	1.00
Socio-economic status						
High	0.27 ± 1.08	26 (14.7)	215 (31.8)	1.00	–	–
Middle	0.64 ± 2.01	23 (13.1)	120 (17.7)	2.74	1.60–4.67	< 0.01
Low	1.65 ± 4.55	127 (72.2)	342 (50.5)	1.75	1.04–2.95	0.03
OHI-S						
Good	1.39 ± 3.60	41 (23.3)	142 (21.0)	1.00	–	–
Fair	0.95 ± 3.62	114 (64.8)	486 (71.8)	1.58	0.83–2.99	0.16
Poor	1.51 ± 3.03	21 (11.9)	49 (7.2)	1.55	0.87–2.74	0.13
Caries						
Caries absent	1.02 ± 3.61	68 (66.7)	609 (81.1)	1.00	–	–
Caries present	1.60 ± 3.22	34 (33.3)	142 (18.9)	2.02	1.26–3.22	0.03

*OR* odds ratio; *C.I* confidence interval

## Discussion

The aim of the study was to assess the impact of DDE on the oral health quality of life of children resident in Ile-Ife and Ibadan, Nigeria. We found that MIH and enamel hypoplasia, with or without caries, had no significant impact on the quality of life of study participants. However, speaking was negatively impacted in children with enamel hypoplasia and caries when compared with children who had enamel hypoplasia but did not have caries. Also, the two significant predictors of oral health quality of life of the study population were socio-economic class and caries: the oral health quality of life of children from the middle and low socio-economic classes, and children with caries was impacted negatively.

The study had some limitations however. The few cases of DDE detected and the low prevalence of caries in the population of children with DDE limited the robustness of the subgroup analysis. Also, prevalence of DDE chosen for the power calculation was high for the study population - higher than the prevalence of DDE identified in prior studies for the population being studied. The use of such a high prevalence implies that the sample size for the study will be less than actually required with implications for under-powering this study. The findings of this study can therefore only be considered an indication of what the probable status of the oral health quality of life of children with DDE could be. An appropriately powered study will give definitive outcomes. In addition, caries was only diagnosed when it affected the dentine. This implies that enamel caries was excluded from the diagnosis of caries in this study thereby leading to an underestimation of the prevalence of caries in the study population. Also, although the results of the chi square tests and logistic regression Goodness of Fit test showed the model was not a good fit, we feel that this may be a type 1 error (incorrect rejection of an acceptable model) due to the low number of observation we had. The hypothesis-testing rationale of this research is more appropriate for testing statistical significance than evaluating the goodness of fit [48]. Despite these limitations, the study provided some insight into the impact of DDE with and without carious lesions, on the oral health quality of life of children in the study environment.

First, a finding of the study suggests that DDE does not affect the overall quality of life just like Vargas-Ferreira and Ardenghi [17] and Arrow [13] observed. However, unlike Vargas Ferreira and Ardenghi [17] who found that children reported significantly higher impact of DDE on functional limitation, we found no significant impact of DDE on any of the eight functional and social items explored.

DDE are often associated with discoloration. The assumption was that the discoloration associated with MIH and enamel hypoplasia may affect the psychological welfare of study participants enough to affect their quality of life. The study suggests that the discoloration associated with these defects is not significant enough to affect the quality of life of children within this age group [49] in this study population. In Columbia, a significantly high number of children with a form of DDE- fluorosis – actually had psychological impact as a result of the discoloration [50]. This raises the question of possible cultural differences in perception of aesthetics and psychological effect of aesthetics on self-welfare [51].

Second, like Arrow [13] and Chukumah et al. [30], we found that caries had a significant negative impact on the oral health quality of life of the study participants. We also found that caries worsened the impact of enamel hypoplasia on the oral health quality of life of children. The study reinforces previous documentations on the deleterious effect of caries on the oral health of affected persons. Caries is known to negatively impact the quality of life in several ways: untreated caries results in pain and aesthetic issues [49] with pain being the most significant factor that impacts negatively on the quality of life [29].

Third, just like a number of researchers had earlier highlighted [52, 53], we also observed that children with lower socio-economic status experienced more negative impact on their oral health-related quality of life, irrespective of the presence of DDE. In the study environment, a child’s socio-economic status does not increase the risk for DDE [30], caries [54] and poor oral hygiene [55]. However, children with low socio-economic status had increased risk for gingivitis [56]. This may be the possible path through which the child’s socio-economic status impacts negatively on the oral health. This postulation however, needs to be explored further.

Finally, the study highlighted a few interesting findings about MIH and enamel hypoplasia. Like multiple other studies, the prevalence of enamel hypoplasia was higher than the prevalence of MIH in the study population [22, 23]. Also, the proportion of children with MIH was highest among children of the high socio-economic class, while the proportion of children with enamel hypoplasia was highest among children with low socio-economic status. Oyedele et al. [20] however demonstrated no association between socio-economic status and MIH. Temilola and Folayan [28] also highlighted that socio-economic status cannot be used as a distinguishing feature for MIH and enamel hypoplasia. Multiple studies had highlighted the association between DDEs such as enamel hypoplasia and socio-economic status – with the prevalence of enamel hypoplasia being higher among children with lower socio-economic status [57]. This study finding therefore concurs with findings from prior studies that established an association between enamel hypoplasia and socio-economic status. However, further studies are required to identify if the child’s socio-economic status can be used as a distinguishing risk factor for MIH and enamel hypoplasia. We also noticed that the prevalence of caries was higher in children with enamel hypoplasia when compared with children with MIH. These findings highlight the need for further studies on MIH and enamel hypoplasia, especially in communities where the prevalence of these lesions are high.

## Conclusion

MIH and enamel hypoplasia do not negatively impact the oral health quality of life of children resident in Southwestern Nigeria. Caries and the socioeconomic status of children were the two factors that had significant impact on the oral health quality of life of children in the study environment. Further studies are however required to explore the similarities and differences in the risk factors and risk indicators for MIH and enamel hypoplasia in the study population.

## Abbreviations

Child-OIDP: Child Oral Impact on Daily performance
DDE: Developmental Defects of the Enamel
LGA: Local Government Area
MIH: Molar-Incisor-Hypomineralisation

## References

[1] Proffit, WR. The development of orthodontic problems. In Contemporary Orthodontics. 2nd edition. Edited by Proffit WR. St Louis: Mosby. 1990:110.

[2] Review of the developmental defects of enamel index (DDE Index). Commission on oral health, research & epidemiology (1992). Report of an FDI working group. Int Dent J.

[3] Farah R, Drummond B, Swain M, Williams S (2010). Linking the clinical presentation of molar incisor hypomineralisation to its mineral density. Int J Paediatr Dent.

[4] Weerheijm KL (2003). Molar incisor hypomineralisation (MIH). Eur J Paediatr Dent.

[5] Garot Elsa, Denis Alice, Delbos Yves, Manton David, Silva Mihiri, Rouas Patrick (2018). Are hypomineralised lesions on second primary molars (HSPM) a predictive sign of molar incisor hypomineralisation (MIH)? A systematic review and a meta-analysis. Journal of Dentistry.

[6] Oyedele TA, Folayan MO, Oziegbe EO. Hypomineralised second primary molars: prevalence, pattern and associated co morbidities in 8- to 10-year-old children in Ile-Ife, Nigeria. BMC Oral Health. 2016;16(1):65. 10.1186/s12903-016-0225-9. 10.1186/s12903-016-0225-9 PMC4893208 27259516

[7] Jälevik B. (2010). Prevalence and Diagnosis of Molar-Incisor-Hypomineralisation (MIH): A systematic review. European Archives of Paediatric Dentistry.

[8] Krishnan R, Ramesh M (2014). Molar incisor hypomineralisation: a review of its current concepts and management. SRM J Res Dent Sci.

[9] Oyedele TA, Folayan MO, Adekoya-Sofowora CA, Oziegbe EO (2015). Co-morbidities associated with molar-incisor hypomineralisation in 8 to 16 year old pupils in Ile-Ife, Nigeria. BMC Oral Health..

[10] Martins-Junior PA, Oliveira M, Marques LS, Ramos-Jorge ML (2012). Untreated dental caries: impact on the quality of life of children of low socioeconomic status. Paediatr Dent.

[11] Bekes K, Hirsch C (2012). What is known about the influence of dentine hypersensitivity on oral health-related quality of life?. Clin Oral Investig.

[12] Al-Zarea BK. Satisfaction with appearance and the desired treatment to improve aesthetics. Int J Dent 2013: 912368. 10.1155/2013/912368 PMC3590633 23509462

[13] Arrow P (2013). Child oral health-related quality of life (COHQoL), enamel defects of the first permanent molars and caries experience among children in Western Australia. Community Dent Health.

[14] Slayton RL, Warren JJ, Kanellis MJ, Levy SM, Islam M (2001). Prevalence of enamel hypoplasia and isolated opacities in the primary dentition. Pediatr.

[15] Kanchanakamol U, Tuongratanaphan S, Tuongratanaphan S (1996). Prevalence of developmental enamel defects and dental caries in rural pre-school Thai children. Community Dent Health.

[16] Lukacs JR (1991). Localized enamel hypoplasia of human deciduous canine teeth: prevalence and pattern of expression in rural Pakistan. Hum Biol.

[17] Vargas-Ferreira F, Ardenghi TM (2011). Developmental enamel defects and their impact on child oral health-related quality of life. Braz Oral Res.

[18] Fotedar S, Sogi GM, Sharma KR (2014). Enamel hypoplasia and its correlation with dental caries in 12 and 15 years old students in Shimla, India. J Indian Assoc Public Health Dent.

[19] Masumo R, Bårdsen A, Astrøm AN (2013). Developmental defects of enamel in primary teeth and association with early life course events: a study of 6-36 month old children in Manyara, Tanzania. BMC Oral Health.

[20] Oyedele TA, Folayan MO, Adekoya-Sofowora CA, Oziegbe EO (2015). Prevalence, pattern and severity of molar incisor hypomineralisation in 8 to 10 year-old children in Ile-Ife, Nigeria. Eur Arch Paediatr Dent.

[21] Temilola DO, Folayan MO, Oyedele TA (2015). The prevalence and pattern of deciduous molar hypomineralization and molar-incisor Hypomineralization in children from a suburban population in Nigeria. BMC Oral Health.

[22] Orenuga OO, Odukoya O (2010). An epidemiological study of developmental defects of enamel in a group of Nigerian school children. Pesq Bras Odontoped Clin Integr João Pessoa.

[23] Temilola DO, Folayan MO, Fatusi O (2014). The prevalence, pattern and clinical presentation of developmental dental hard-tissue anomalies in children with primary and mix dentition from Ile-Ife, Nigeria. BMC Oral Health..

[24] Salako N, Adenubi J (1984). Chronologic enamel hypoplasia. Tropical Dental Journal.

[25] Sawyer DR, Taiwo EO, Mosadomi A (1984). Oral anomalies in Nigerian children. Community Dent Oral Epidemiol.

[26] Popoola BO, Onyejaka NK, Folayan MO (2017). Prevalence of developmental dental hard-tissue anomalies and association with caries and oral hygiene status of children in southwestern, Nigeria. BMC Oral Health.

[27] Adenubi J (1980). Dental status of 4 and 5 year old children in Lagos private schools. Niger Dent J.

[28] Temilola Oluwaseyi Dada, Folayan Morenike Oluwatoyin (2015). Distinguishing predisposing factors for enamel hypoplasia and molar-incisor hypomineralization in children in Ile-Ife, Nigeria. Brazilian Journal of Oral Sciences.

[29] Oziegbe EO, Esan TA, Adesina BA (2012). Impact of oral conditions on the quality of life of secondary schoolchildren in Nigeria. J Dent Child (Chic).

[30] Chukwumah NM, Folayan MO, Oziegbe EO, Umweni AA (2016). Impact of dental caries and its treatment on the quality of life of 12- to 15-year old adolescents in Benin, Nigeria. Int J Paed Dent.

[31] Oziegbe EO, Esan TA, Oyedele TA (2014). Brief communication: emergence chronology of permanent teeth in Nigerian children. Am J Phys Anthropol.

[32] Enwonwu CO (1976). Influence of socio-economic conditions on dental development in Nigerian children. Arch Oral Biol.

[33] Benson J, Borman G (2010). Family, neighborhood, and school settings across seasons: when do socioeconomic context and racial composition matter for the reading achievement growth of young children?. Teach Coll Rec.

[34] National Bureau of Statistics. 2006 Population Census. 2006. Internet: http://www.nigerianstat.gov.ng/nbsapps/Connections/Pop2006.pdf. Accessed 4 July, 2011.

[35] Secretariat. Commission, Statistics section. Ibadan, Nigeria. 2001.

[36] Cochran WG (1977). Sampling Techniques.

[37] Abanti J, Carvalho TS, Bonecker M, Ortega AO, Ciamponi AL, Raggio RP (2012). Parental reports of the oral health related quality of life of children with cerebral palsy. BMC Oral Health..

[38] Gherunpong S, Tsakos G, Sheiham A (2004). Developing and evaluating an oral health-related quality of life index for children; the CHILD-OIDP. Community Dent Health.

[39] World Health Organization (1997). Oral health survey-basic method.

[40] Krapp K. Dental Indices. Encyclopedia of Nursing & Allied Health. Ed. Vol. 2. Gale Cengage. eNotes.com. 2002. http:// www. enotes. com/ dental-indices-reference/. Assessed 2 Jan, 2012.

[41] Seow WK, Ford D, Kazoullis S, Newman B, Holcombe T (2011). Comparison of enamel defects in the primary and permanent dentitions of children from a low-fluoride district in Australia. Pediatric Dent.

[42] Greene JC, Vermillion JR (1964). The simplified oral hygiene index. J Am Dent Assoc.

[43] Weerheijm KL, Duggal M, Mejare I (2003). Judgement criteria for MIH in epidemiologic studies: summary of the European meeting on MIH held in Athens. Eur J Paediatr Dent.

[44] Folayan M, Idehen E, Ufomata D (2003). The effect of socio-demographic factors on dental anxiety in children seen in a sub-urban Nigerian hospital. Int J Paediatr Dent.

[45] Nakayama N. (2016). The Relationship Between Linear Enamel Hypoplasia and Social Status in 18th to 19th Century Edo, Japan. International Journal of Osteoarchaeology.

[46] Petersen PE (2005). Sociobehavioural risk factors in dental caries—international perspectives. Community Dent Oral Epidemiol.

[47] Holst D, Schuller AA, Aleksejuniené J, Eriksen HM (2001). Caries in population—a theoretical, causal approach. Eur J Oral Sci.

[48] Marsh HW, Hau K-T, Wen Z (2004). In search of Golden rules: comment on hypothesis-testing approaches to setting cutoff values for fit indexes and dangers in overgeneralizing Hu and Bentler's (1999) findings. Struct Equ Model Multidiscip J.

[49] Peres KG, Latorre Mdo R, Peres MA, Traebert J, Panizzi M (2003). Impact of dental caries and dental fluorosis on 12-year-old schoolchildren's self-perception of appearance and chewing. Article in Portuguese Cad Saude Publica.

[50] Tellez M, Santamaria RM, Gomez J, Martignon S (2012). Dental fluorosis, dental caries, and quality of life factors among schoolchildren in a Colombian fluorotic area. Community Dent Health.

[51] Osborne H (1986). Aesthetic experience and cultural value. The Journal of Aesthetics and Art Critic.

[52] Leal SC, Bronkhorst EM, Fan M, Frencken JE (2012). Untreated cavitated dentine lesions: impact on children’s quality of life. Caries Res.

[53] Paula JS, Meneghim MC, Pereira AC, Mialhe FL (2015). Oral health, socio-economic and home environmental factors associated with general and oral health related quality of life and convergent validity of two instruments. BMC Oral Health..

[54] Kumar S, Kroon J, Lallo R (2014). A systematic review of the impact of parental socioeconomic status and home environmental characteristics on children’s oral health related quality of life. Health Qual Life Outcomes.

[55] Folayan MO, Kolawole KA, Oziegbe EO, Oyedele T, Oshomoji OV, Chukwumah NM, Onyejaka N (2015). Prevalence, risk factors and predictors of early childhood caries in children resident in sub-urban Nigeria. BMC Oral Health..

[56] Agbaje HO, Kolawole KA, Folayan MO, Onyejaka N, Oziegbe EO, Oyedele TA, Chukwumah N, Oshomoji OV (2016). Digit sucking, age, gender and socioeconomic status as determinants of oral hygiene status and gingival health of children in suburban Nigeria. J Periodontol.

[57] Vargas-Ferreira F, Salas MM, Nascimento GG (2015). Association between developmental defects of enamel and dental caries: a systematic review and meta-analysis. J Dent.

